# Fecal microbiota transplantation against intestinal colonization by extended spectrum beta-lactamase producing *Enterobacteriaceae*: a proof of principle study

**DOI:** 10.1186/s13104-018-3293-x

**Published:** 2018-03-22

**Authors:** Ramandeep Singh, Pieter F. de Groot, Suzanne E. Geerlings, Caspar J. Hodiamont, Clara Belzer, Ineke J. M. ten Berge, Willem M. de Vos, Frederike J. Bemelman, Max Nieuwdorp

**Affiliations:** 1 Renal Transplant Unit, Division of Nephrology, Academic Medical Centre, Room A3-273, PO box 22660, 1100 DD Amsterdam, The Netherlands; 2 Division of Vascular Medicine, Academic Medical Centre, Room F4-256, PO box 22660, 1100 DD Amsterdam, The Netherlands; 3 Division of Infectious Diseases, Center of Infection and Immunity Amsterdam (CINIMA), Academic Medical Centre, Amsterdam, The Netherlands; 4 Department of Medical Microbiology, Academic Medical Centre, Amsterdam, The Netherlands; 5 Department of Microbiology, Wageningen University, Wageningen, The Netherlands; 6 Department of Internal Medicine, VU University Medical Center, Amsterdam, The Netherlands; 7 ICaR-VU, VU University Medical Center, Amsterdam, The Netherlands; 8 Wallenberg Laboratory, Sahlgrenska Hospital, University of Gothenburg, Gothenburg, Sweden

**Keywords:** Multidrug resistance microorganisms, Microbiota, ESBL, Fecal microbiota transplantation

## Abstract

**Objective:**

Infections with multidrug-resistant microorganisms are associated with increased hospitalization, medication costs and mortality. Based on our fecal microbiota transplantation (FMT) experience for *Clostridium difficile* infection, we treated 15 patients carrying ESBL-producing *Enterobacteriaceae* (ESBL-EB) with FMT. Seven patients underwent a second FMT after 4 weeks when ESBL-EB remained, amounting to a total number of 22 transplants. The objective was decolonization of ESBL-EB.

**Results:**

Three out of fifteen (20%) patients were ESBL-negative at 1, 2 and 4 weeks after the first transplant, while six out of 15 (40%) were negative after the second transplant. Comparison of fecal microbiota at baseline and 4 weeks after FMT revealed restoration of microbial diversity after FMT and a microbial shift towards donor composition. Finally, we suggest several possible factors of response to therapy, such as donor-recipient microbiota match and number of FMTs. Therefore, FMT can be an effective treatment in patients carrying ESBL-EB. Response may be determined by microbiota composition and number of FMT procedures.

*Trial registration* ISRCTN ISRCTN48328635 Registered 11 October 2017, retrospectively registered

**Electronic supplementary material:**

The online version of this article (10.1186/s13104-018-3293-x) contains supplementary material, which is available to authorized users.

## Introduction

The increased prevalence of antimicrobial-resistant microorganisms is one of the most important medical challenges faced by the worldwide infectious disease community [1]. Antibiotic use is the main driving force behind the development of antimicrobial resistance. Extended-spectrum beta lactamase is a group of enzymes that can hydrolyse penicillin and cephalosporin group antibiotics, rendering these antibiotics ineffective. The presence of extended spectrum beta lactamase (ESBL) genes in *Enterobacteriaceae* leads to delayed effective treatment of infections with these multiresistant microorganisms, which is associated with higher mortality, longer hospital admission and higher medical expenditures [2–4]. ESBL producers can be cultured from any human body site, however most *E. coli* causing urinary tract infections (UTI) derive from the patient’s gut microbiota [5]. Since systemic antimicrobial exposure may contribute to further development of antimicrobial resistance, antimicrobial-free ESBL decolonization schemes are preferred. In this regard, fecal microbiota transplantation (FMT), in which the intestinal microbiota of a healthy donor is infused through a gastroduodenal tube may be considered. We and others have previously shown that FMT can successfully treat recurrent *Clostridium difficile* infection (CDI), with an overall cure rate of more than 90% [6, 7]. To date, over eight case reports and three case series have been published on successful FMT for MDRO decolonization, varying in treatment method, amount of feces administered, number of administrations, preparation (antibiotics and/or lavage) and targeted pathogen [8, 9]. Currently several clinical trials are underway to evaluate effectivity of FMT against a range of MDRO (https://clinicaltrials.gov; NCT02312986, NCT02543866, NCT02461199, NCT02390622, NCT02472600). Only one is a randomized trial with a ‘no treatment’ group as control group, while the others are prospective cohort studies with a single treatment group. In this pilot study we aimed to investigate decolonization of ESBL-producing bacteria using FMT.

## Main text

### Methods

#### Patient and donor selection and FMT procedure

Out of 155 rectal swab ESBL-positive patients in our hospital between 2012 and 2014, those with a life-expectancy of at least 6 months were selected. Patients’ clinical data was obtained from their hospital case files. Exclusion criteria were negative rectal swab upon retesting, food allergies and severe immunodeficiency. This resulted in 75 patients that were potentially eligible for FMT. Of these, patients 22 declined to participate, twenty had a negative rectal swab culture upon retesting, eleven could not fulfill the logistic requirements of the study [were not physically or practically able to appear at the study visits (10), or had plans to travel abroad during the study(1)], six were severely immunocompromised (CD4 count below 200 cells/µl) or had a life-expectancy of less than 3 months and one had a food allergy. The remaining fifteen patients agreed to undergo FMT. Of these, five (33%) were renal transplant recipients using immunosuppressive drugs. Thirteen patients suffered from recurrent urinary tract infections. Other patient characteristics and comorbidity can be found in Table 1. Of 22 pre-FMT cultures, 19 showed an ESBL-producing *E. coli*. The other three showed *Klebsiella pneumoniae* (Table 1).

**Table 1:** Patient characteristics

**#**	Age	Sex	BMI (kg/m^2^)^a^	Comorbidity	ESBL-Producer	ESBL-neg.^b^ after 1st FMT^c^	ESBL-neg. after 2nd FMT	Donor FMT 1	Donor FMT 2
1	58	M	19	ESRD^d^, PD^e^, CVD^f^	*E. coli* ^g^	Y	–	1	–
2	47	M	27	Tetraplegia, rUTI^h^	*E. coli*	N	–	1	–
3	65	M	25	Renal Tx^j^, rUTI	*E. coli*	N	N	1	1
4	61	M	24	rUTI	*K. p* ^j^	N	–	1	–
5	29	F	35	rUTI	*E. coli*	N	Y	1	2
6	56	F	28	RUTI	*E. coli*	N	N	1	2
7	70	F	28	Renal Tx, rUTI	*K. p, E. coli*	N	N	1	2
8	59	F	20	Renal Tx, rUTI, HBV^k^	*E. coli*	Y	–	1	–
9	61	F	28	rUTI	*E. coli*	N	Y	1	1
10	57	F	26	ESRD, rUTI	*E. coli*	N	–	2	–
11	76	F	23	rUTI	*E. coli*	Y	–	2	–
12	70	M	24	Renal Tx, rUTI, T2D^l^	*E. coli*	N	–	1	–
13	59	F	29	Renal Tx	*K. p*	N	N	1	1
14	58	F	36	rUTI	*E. coli*	N	Y	1	2
15	21	F	24	rUTI	*E. coli*	N	–	1	–

^a^Body mass index, ^b^ extended-spectrum beta lactamase producer-negative, ^c^ fecal microbiota transplantation, ^d^ end-stage renal disease, ^e^ peritoneal dialysis, ^f^ cardiovascular disease, ^g^ *Escherichia coli*, ^h^ recurring urinary tract infections, ^i^ transplant, ^j^ *Klebsiella pneumoniae*, ^k^ Hepatitis B virus, ^l^ type 2 diabetes. Patient 7 had (only) *K. pneumoniae* before the 1st FMT and (only) *E. coli* before the second FMT

Fecal microbiota transplantation and screening of donors were performed according to the FMT protocol used as previously published [7] (also refer to the Additional file 1: Methods). No antibiotics were administered before or during FMT. When the patient remained ESBL-positive at 1, 2 and 4 weeks after the first FMT, a second treatment was proposed. Two male donors were used for all transplantations: donor 1 was the default donor and donated 16 times. Donor 2 was used when donor 1 was unavailable (twice) or after a failed attempt if he was available (four times) (see Table 1 ‘patient characteristics’). Written informed consent was obtained from all participants. The study was approved by the local ethics committee and conducted at the Academic Medical Center (Amsterdam), in accordance with the Declaration of Helsinki (updated version 2013). ESBL-EB rectal and urine surveillance cultures were taken at week 1, 2, and 4 after FMT. ESBL-EB surveillance culture procedures, FMT procedures and donor selection are discussed in the Additional file 1: Methods.

#### Microbiota analyses

Fecal samples were taken at baseline and 4 weeks after FMT. Microbiota were analyzed from these samples in Wageningen University by human intestinal tract (HIT-) chip, a custom-made microarray containing roughly 5500 specific oligonucleotide probes that cover over 1000 intestinal phylotypes, which is used for the high-throughput profiling of the fecal microbiota [10]. For a more detailed description, please refer to the Additional file 1: Methods.

#### Statistical analyses

For the microbiota plots we have performed a principal component analyses, which is a commonly used statistical procedure used for graphical representation of microbiota compositional differences between samples, usually depicted as distances between dots in a two-dimensional space showing only principal component 1 and 2 (on the x- and y-axis), which by definition explain most of the variance. This was done in R-studio (version 0.99.903). Comparison between microbiota at baseline and 4 weeks after FMT was done using Wilcoxon’s signed rank test for each HITchip phylotype. P values were corrected for multiple testing using ‘qvalue’ package in R. Comparison between responder and nonresponder microbiota at baseline and 4 weeks after FMT were done with Wilcoxon’s signed rank test.

### Results

#### Fecal microbiota transplantation

**Fig. 1:**
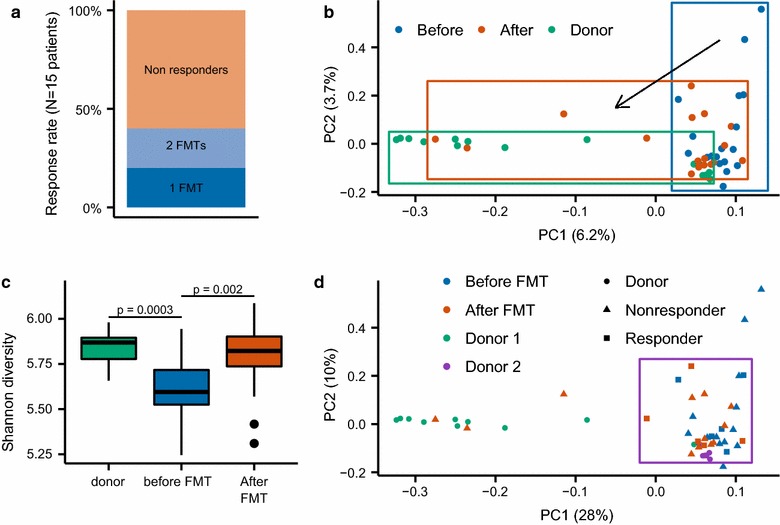
**a** Percentage of responders after one and two FMTs. **b** PCA-plot showing microbiota composition of donors and recipients before and after FMT. **c** Microbial diversity **d** Microbiota of responders and non-responders. The black square encloses all responders

In total fifteen patients underwent an FMT procedure, of whom seven underwent a second FMT. After the first FMT procedure, decolonization was successful in three patients (20%) at all timepoints (1, 2 and 4 weeks follow-up), except for subject 1 who showed a delayed response and became negative at week 2 (Additional file 2: Table S4). Patients who remained ESBL-EB negative on follow-up were named ‘responders’. The twelve non-responders remained ESBL-EB positive on all timepoints. Seven out of twelve non-responders underwent a second FMT, three of which led to decolonization at all timepoints (1, 2 and 4 weeks) after the second FMT, resulting in an overall eradication success rate of 40% Additional file 2: Table S4 and Fig. 1a). Patient characteristics are summarized in Table 1. Subsequent analyses showed that ESBL eradication was associated with having had two FMTs (3/7 or 43% success rate) instead of one FMT (3/15 or 20% success rate) as well as FMT donor specific treatment effects (donor 2 had a 50% success rate (3 out of 6) versus 19% of donor 1 (3 out of 16)). Moreover, immunocompromised state (e.g. patients with a kidney transplant) was associated with a lower success rate (1/5 or 20%). It should be stressed that these findings are not statistically significant and can thus be merely hypothesis-raising. Patient characteristics of responders and non-responders are reported in Additional file 3: Table S1, success rate and patient characteristics per transplantation are shown in Additional file 4: Table S2. Finally and importantly, no side effects besides mild discomfort and temporary loose stools (< 24 h) were reported after FMT.

#### Fecal microbiota analysis

At 4 weeks after FMT, a general shift in fecal microbiota composition towards donor microbial composition was seen in the patients, depicted in Fig. 1b as a shift to the left along principal component 1 (PC1) and downward along principal component 2 (PC2). Overall, fecal microbial diversity (Shannon’s diversity index) also changed significantly after FMT (Fig. 1c).

Although fecal microbiota composition in responders did not form a separate cluster from the non-responder microbiota in the principal component analysis (Fig. 1d), responder microbiota clustered more tightly (Fig. 1d, inside the purple square). Interestingly, responder microbiota composition resembled donor 2 composition more than donor 1 composition (Fig. 1d) which coincides with the observation that donor 2 seemed the more effective of the two.

In total, 664 bacterial taxa changed significantly after FMT (Wilcoxon’s, adjusted q-value < 0.05). Between responders and non-responders 21 taxa were significantly different (Wilcoxon’s, *p* value < 0.05) at 4 weeks after FMT, seven of which had also changed significantly and more than twofold in abundance after FMT (Additional file 5: Figure S1). At baseline, six species were significantly different between responders and nonresponders (Additional file 6: Table S3).

### Discussion

Decolonization of ESBL-EB with antibiotic regiments may be achieved, but is often followed by recolonization [11]. In contrast, FMT may decolonize while preventing relapse and work in synergy with antibiotic regimens, as it does in the treatment of recurrent CDI [12]. In our small explorative study, fecal microbiota transplantation was explored as a novel treatment against colonization by multi-drug-resistant microorganisms (MDRO).

As mentioned in the introduction, several studies have been published on the subject. Noteworthy is a recent case series in which 60% of 25 patients with blood disorders were decolonized from antibiotic-resistant pathogens when sampled 1 month after FMT [13]. While this study features a larger sample size, our study sampled earlier after FMT and at several time points, minimizing the chance of spontaneous clearance as explanation for success. Furthermore, none of our subjects used antibiotics concurrently with FMT treatment.

FMT is already successfully being applied for *Clostridium difficile* infection (CDI), for which it is highly effective [14]. For other indications response rates seem modest thus far. Therefore, factors that predict response or non-response need to be identified, requiring a more detailed analytic approach. In the prospect of larger trials, our small uncontrolled study shows several interesting clues.

Interestingly, a recent case series has shown that five out of five patients cleared MRSA after nasojejunal administration of FMT using three consecutive FMTs [15]. Although it should be noted that in this study pre-treatment antibiotics (vancomycin) was used and pathogen type was different, its findings are in line with our finding that repeat-FMT leads to a higher success rate. It should be noted however, that optimal dose, frequency and route of administration of FMT in ESBL is still unclear.

Our study shows that FMT in ESBL-EB colonization restores bacterial diversity, similar to FMT in CDI [7]. To further dissect driving mechanisms, we studied changes in fecal microbial signatures associated with response to FMT and observed that microbiota in responders (as opposed to microbiota in non-responders) cluster in a relatively tight formation and more closely resemble the microbiota of donor 2 (Fig. 1d). We therefore hypothesize that improved donor-recipient match improves FMT success rate and may explain the higher success rate of donor 2 in our study (3/6 vs. 3/16 of donor 1). Finally, seven bacterial taxa had at the same time changed significantly after FMT and were significantly different between responders and non-responders (Additional file 5: Figure S1), including several known butyrate producers.

Thus far, FMT studies generally focus on bacterial composition. It is often postulated, for CDI but also for ESBL, that healthy donor bacteria simply restore diversity and cure the disease by outcompeting pathogens. However, although microbiota composition changed markedly and diversity increased after FMT in our study, no distinction between responders and non-responders could be made based on these factors. Furthermore, a recent uncontrolled study convincingly disqualifies direct transfer of bacteria as the curative mechanism in CDI by showing sterile fecal filtrates (obtained by filtration of a pre-processed fecal solution through a 0.2 µm filter) derived from donor feces cure CDI just as well (five out of five patients) [16]. This emphasizes the importance of other fecal solutes, such as bacterial products, bacteriophages or other immune system-provoking agents in explaining how FMT can lead to disease resolution.

### Conclusion

In conclusion we have shown an efficacy of (repeat-) FMT in ESBL decolonization of 6/15 (40%). Our results suggest repeat FMT increases treatment effectivity. Possibly, donor choice, patient characteristics and donor-patient match also play a role. Our study shows microbial diversity restoration after FMT, without relation to response rate. Several microbes are associated with response. In the future, larger randomized-controlled studies should confirm these findings. These analyses should ideally not be limited to characterization of gut bacteria, but also include other active agents in the fecal solution.

## Limitations

Our study has some important limitations. The first limitation is that it lacks a control group. It can be postulated that not FMT but rather spontaneous clearance has caused decolonization in our ‘responders’. Indeed, spontaneous clearance of MDRO in previously hospitalized patients after discharge is common (about 20–30% of cases) [17, 18]. However, our population consisted of outpatients with prolonged colonization (months to years) in a home setting. Since there was only 1 week between pre-FMT sampling and post-FMT sampling in which this spontaneous clearance could have happened, we dare say that we expect the contribution of spontaneous clearance on our treatment success rate is modest. Secondly, another limitation of our study is the small study group, therefore the factors of success that we identified were not statistically significant. Thirdly, longer follow-up than 4 weeks would provide valuable information on whether FMT protects from relapse after new antibiotic courses. And finally, our population consisted of patients with various and often severe medical conditions, among which several renal transplant subjects. Therefore it is doubtful whether our data can be extrapolated to the general population. That being said, our population is representative in the sense that it is a patient group suffering from recurrent urinary tract infections with ESBL-EB.

## Additional files

**Additional file 1: Methods.** Additional materials and methods.

**Additional file 2: Table S4.** Culture results in decolonized subjects. Rectal and urine cultures at follow-up (baseline, 1, 2 and 4 weeks after FMT).

**Additional file 3: Table S1.** Characteristics of responders vs nonresponders. Characteristics of responders vs nonresponders.

**Additional file 4: Table S2.** Characteristics of successful FMTs vs unsuccessful FMTs. Characteristics of successful FMTs vs unsuccessful FMTs.

**Additional file 5: Figure S1.** Heat map of change in responders vs nonresponders. Heatmap showing fold change in microbiota abundance of significantly different species between responders and non-responders before versus after FMT. FMTs are shown, therefore patients who have received 2 FMTs are shown twice. Faecalibacterium 1 and 2 are both subgroups of the Faecalibacterium genus. Faecalibacterium 1 is the genus in the strict sense, whereas the Faecalibacterium 2 group includes uncultured bacteria related to the phylotypes Eldhufec289, Eldhufec276 and Eldhufec259.

**Additional file 6: Table S3.** Abundances of taxa in responders vs nonresponders. Relative abundances of significantly different taxa (only < 0.9 or > 1.1 fold shown) between responders and nonresponders at baseline. Median relative abundance (percentage) is shown, also the ratio between the medians of responders and nonresponders and the p-value are given.

## Abbreviations

CDI: *Clostridium difficile* infection
ESBL: extended spectrum beta lactamase
EB: *Enterobacteriaceae*
ESBL-EB: extended spectrum beta lactamase producing enterobacteriaceae
FMT: fecal microbiota transplantation
HIT-chip: human intestinal tract chip
HIV: human immunodeficiency virus
MALDI-TOF: matrix assisted laser desorption/ionisation time-of-flight analyzer
MIC: minimum inhibitory concentration
MDRO: multi-drug-resistant microorganisms
MS: mass spectrometer
PC: principal component
UTI: urinary tract infections
