# Supplementary material for: Fecal microbiota transplantation against intestinal colonization by extended spectrum beta-lactamase producing Enterobacteriaceae: a proof of principle study
Source: BMC Res Notes. 2018 Mar 22;11:190. doi: 10.1186/s13104-018-3293-x (PMC5863815; doi:10.1186/s13104-018-3293-x)
Supplement: Supplementary file 1 — Additional file 1: Methods. Additional materials and methods. [file 13104_2018_3293_MOESM1_ESM.doc]

**Supplementary methods**

Fecal transplant procedures

Each patient underwent complete colon lavage with macrogol (Klean-Prep) one day prior to FMT. At the day of FMT and after an overnight fast, the patient arrived at the hospital for the placement of a nasoduodenal tube. No antibiotics were given prior or during FMT. Between 200 and 300 grams of donor feces was processed within two hours after production. It was diluted in 500 ml saline and filtered through unfolded cotton gauzes. The filtrate was administered through to the patient through the duodenal tube using ten 50cc syringes in a time course of around 30 minutes. The ‘proximal route’ (i.e. via duodenal tube) was used because we have experience with this technique in our center. A possible advantage is that the fecal solution reaches the entire intestinal tract, a disadvantage is that when the patient vomits there is a risk of aspiration. Therefore, difficulty swallowing is a relative contra-indication for fecal transplantation by duodenal tube. In general the technique is well-tolerated by our patients. For clostridium difficile, both techniques (duodenal route and through enema) seem equally effective.

Donor selection

Healthy adult male donors were selected by advertisement from the population in the region of Amsterdam. Medication use, recent (<3 months) use of antibiotics or proton pump inhibitors, complaints of irritable bowel syndrome and a first degree relative with certain microbiota-associated diseases (e.g. colon cancer before 65 years of age, several autoimmune disorders) were exclusion criteria for potential donors. Donors were thoroughly screened for ESBL and for communicable diseases (blood tests for HIV, hepatitis A, B and C, syphilis, human T-lymphotrophic virus I and II, Epstein-Barr virus, cytomegalovirus, *Strongyloides* and *Entamoeba*. In the feces cultures for *Shigella*, *Salmonella*, *Yersinia*, *Campylobacter* and *E.coli* O157:H7 was performed, PCR for adenovirus, astrovirus, enterovirus, norovirus, parechovirus, rotavirus and sapovirus was done and a dual feces test was performed to screen for pathogenic parasites.

ESBL-EB surveillance cultures

Fecal samples were incubated overnight in non-selective tryptic soy broth. The next day, the broth was plated on a chromID ESBL plate (bioMérieux, Marcy I'Etoile, France), a selective chromogenic agar medium where species can be easily distinguished based on morphology and colour (e.g. E. coli colonies appear pink and colonies of Klebsiella/Enterobacter/Serratia species appear blue/green). ChromID ESBL plates were incubated for 18 hours at 37°C under aerobic conditions. Each colony type growing on ChromID ESBL was identified by MALDI-TOF MS using the Bruker Biotyper (Bruker Daltonics, Germany). Antimicrobial susceptibility testing was performed using the VITEK2 system (bioMérieux). If the isolate proved to have an MIC >1 mg/L for cefotaxime or ceftazidime, ESBL-production was confirmed phenotypically using the combination disk diffusion test, according to the guidelines of the Dutch Society of Medical Microbiology (NVMM), available online at [http://www.nvmm.nl/system/files/2012.11.15%20richtlijn%20BRMO%20(version%202.0)%20-%20RICHTLIJN.pdf](http://www.nvmm.nl/system/files/2012.11.15 richtlijn BRMO (version 2.0) - RICHTLIJN.pdf)). Briefly, a bacterial suspension of 0.5 McFarland was inoculated on Mueller-Hinton agar. Cephalosporin disks were applied and plates were incubated for 18 hours at 37°C under aerobic conditions. For *E. coli* and *Klebsiella* spp, the difference between cefotaxime and cefotaxime + clavulanic acid and between ceftazidime and ceftazidime + clavulanic acid was evaluated. The test was positive if the inhibition zone was ≥5 mm larger with than without clavulanic acid. The procedure for urine samples was identical, with one exception: no broth was used, but samples were plated directly on a chromID ESBL plate.

Microbiota analyses

Fecal samples were taken at baseline and 4 weeks after FMT. Samples were taken at home in a non-sterile container and stored at the study visit in -80 degrees until DNA-extraction for HIT-chip. Two participants failed to turn in fecal samples for microbiota sequencing. Furthermore, two baseline samples were not turned in (one was a responder and one a nonresponder), so they could not be depicted in supplementary figure 1. Microbiota were analyzed from these samples in Wageningen University by human intestinal tract (HIT-)chip, a custom-made microarray containing roughly 5500 specific oligonucleotide probes that cover over 1000 intestinal phylotypes, which is used for the high-throughput profiling of the fecal microbiota. Resulting relative abundance tables were loaded into R for statistical analysis.

Statistical analyses

For the microbiota plots we have performed a principal component analyses, which is a commonly used statistical procedure used for graphical representation of microbiota compositional differences between samples, usually depicted as distances between dots in a two-dimensional space showing only principal component 1 and 2 (on the x- and y-axis), which by definition explain most of the variance. This was done in R-studio (version 0.99.903). Comparison between microbiota at baseline and four weeks after FMT was done using Wilcoxon’s signed rank test for each HITchip phylotype. P-values were corrected for multiple testing using ‘qvalue’ package in R. Comparison between responder and nonresponder microbiota at baseline and four weeks after FMT were done with Wilcoxon’s signed rank test. Alpha diversity was calculated using Shannons diversity index in R, indices were compared between time points using Wilcoxon’s test.
