# Supplementary material for: Fecal microbiota transplantation against intestinal colonization by extended spectrum beta-lactamase producing Enterobacteriaceae: a proof of principle study
Source: BMC Res Notes. 2018 Mar 22;11:190. doi: 10.1186/s13104-018-3293-x (PMC5863815; doi:10.1186/s13104-018-3293-x)
Supplement: Supplementary file 2 — Additional file 2: Table S4. Culture results in decolonized subjects. Rectal and urine cultures at follow-up (baseline, 1, 2 and 4 weeks after FMT). [file 13104_2018_3293_MOESM2_ESM.doc]

**Supplementary table 4**

| Subject # | Culture site | Timepoints | | | | Re-FMT |
| --- | --- | --- | --- | --- | --- | --- |
|  |  | Pre-FMT | Week 1 | Week 2 | Week 4 |  |
| 1 | Urine:  Rectal: | N/A  ESBL E. coli | N/A  ESBL E. coli | N/A  NEG | N/A  NEG |  |
| 8 | Urine:  Rectal: | ESBL E. coli  ESBL E. coli | NEG  NEG | NEG  NEG | NEG  NEG |  |
| 11 | Urine:  Rectal: | NEG  ESBL E. coli | P. mirabilis  NEG | P. mirabilis  NEG | NEG  NEG |  |
| 5 | Urine:  Rectal: | ESBL E. coli ESBL E. coli | ESBL E. coli ESBL E. coli | ESBL E. coli ESBL E. coli | ESBL E. coli ESBL E. coli | NEG at 1,2 and 4 weeks NEG at 1,2 and 4 weeks |
| 9 | Urine:  Rectal: | ESBL E. coli  ESBL E. coli | ESBL E. coli  ESBL E. coli | ESBL E. coli  ESBL E. coli | ESBL E. coli  ESBL E. coli | Non-ESBL producers  NEG at 1,2 and 4 weeks |
| 14 | Urine:  Rectal: | NEG  ESBL E. coli | NEG  ESBL E. coli | NEG  ESBL E. coli | NEG  ESBL E. coli | NEG at 1,2 and 4 weeks  NEG at 1,2 and 4 weeks |

**Supplementary table 4: Culture results in decolonized subjects**

#: number, FMT: fecal microbiota transfer, N/A: not applicable, ESBL E. coli: beta lactamase producing Escherichia coli, NEG: negative, P: Proteus.
