# Supplementary material for: Fecal microbiota transplantation against intestinal colonization by extended spectrum beta-lactamase producing Enterobacteriaceae: a proof of principle study
Source: BMC Res Notes. 2018 Mar 22;11:190. doi: 10.1186/s13104-018-3293-x (PMC5863815; doi:10.1186/s13104-018-3293-x)
Supplement: Supplementary file 3 — Additional file 3: Table S1. Characteristics of responders vs nonresponders. Characteristics of responders vs nonresponders. [file 13104_2018_3293_MOESM3_ESM.doc]

Supplementary table 1

|  | **Responders** | **Non-responders** |
| --- | --- | --- |
| **No. of patients** | 6 | 9 |
| **Age (mean, years)** | 56,9 | 56,5 |
| **Males (%)** | 1/6 (17%) | 4/9 (44%) |
| **BMI^1^ (mean, kg/m²)** | 26.8 | 26.1 |
| **second FMT^2^ (%)** | 3/6 (50%) | 4/9 (44%) |
| **1^st^ FMT by donor 1 (%)** | 5/6 (85%) | 8/9 (89%) |
| **2^nd^ FMT by donor 1 (%)** | 1/3 (33%) | 2/4 (50%) |
| **Renal Tx^3^ recipients (%)** | 1/6 (17%) | 4/9 (44%) |

**Supplementary table 1: characteristics of responders vs non-responders**

**^1^**body mass index, ^2^fecal microbiota transplantation, **^3^**transplant
