# Supplementary material for: Fecal microbiota transplantation against intestinal colonization by extended spectrum beta-lactamase producing Enterobacteriaceae: a proof of principle study
Source: BMC Res Notes. 2018 Mar 22;11:190. doi: 10.1186/s13104-018-3293-x (PMC5863815; doi:10.1186/s13104-018-3293-x)
Supplement: Supplementary file 4 — Additional file 4: Table S2. Characteristics of successful FMTs vs unsuccessful FMTs. Characteristics of successful FMTs vs unsuccessful FMTs. [file 13104_2018_3293_MOESM4_ESM.doc]

Supplementary table 2

|  | **Successful** | **Unsuccessful** |  |
| --- | --- | --- | --- |
| **No. of FMTs^1^ (n)** | 6 | 16 | |
| **Patient age (mean, years)** | 57.3 | 58.2 | |
| **Male patients (%)** | 1/6 (17%) | 5/16 (31%) | |
| **Patient BMI^2^ (mean, kg/m²)** | 26.8 | 27.8 | |
| **No. of repeat FMTs (%)** | 3/6 (50%) | 4/16 (25%) | |
| **FMTs by donor 1 (%)** | 3/6 (50%) | 13/16 (81%) | |
| **FMTs by donor 2 (%)** | 3/6 (50%) | 3/16 (19%) | |
| **Renal Tx^3^ recipients (%)** | 1/6 (17%) | 7/16 (44%) | |

**Supplementary table 2: characteristics of successful FMTs vs unsuccessful FMTs**

**^1^**fecal microbiota transplantation, **^2^**body mass index, **^3^**transplant
