# Supplementary figures and images for: Fecal microbiota transplantation against intestinal colonization by extended spectrum beta-lactamase producing Enterobacteriaceae: a proof of principle study
Source: BMC Res Notes. 2018 Mar 22;11:190. doi: 10.1186/s13104-018-3293-x (PMC5863815; doi:10.1186/s13104-018-3293-x)

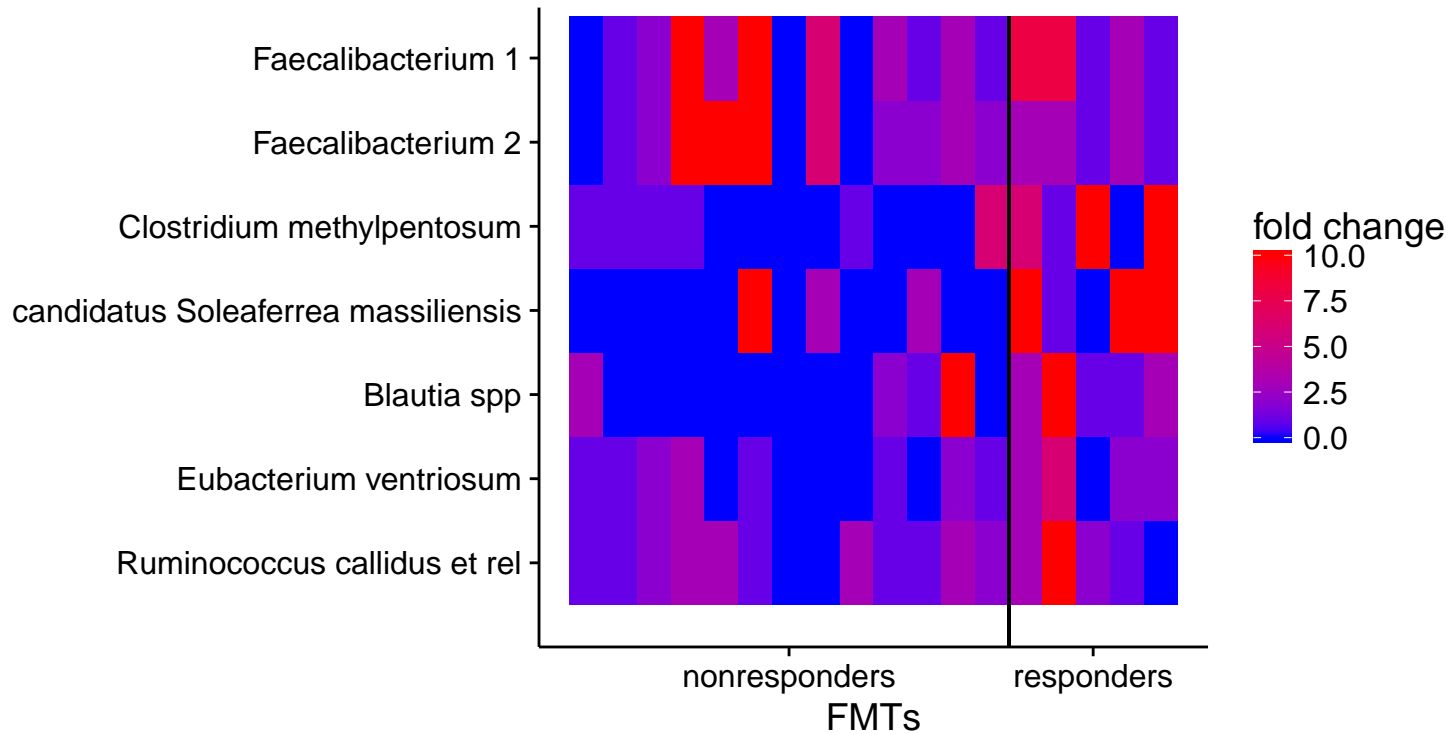

Supplement: Supplementary file 5 — Additional file 5: Figure S1. Heat map of change in responders vs nonresponders. Heatmap showing fold change in microbiota abundance of significantly different species between responders and non-responders before versus after FMT. FMTs are shown, therefore patients who have received 2 FMTs are shown twice. Faecalibacterium 1 and 2 are both subgroups of the Faecalibacterium genus. Faecalibacterium 1 is the genus in the strict sense, whereas the Faecalibacterium 2 group includes uncultured bacteria related to the phylotypes Eldhufec289, Eldhufec276 and Eldhufec259. [file 13104_2018_3293_MOESM5_ESM.pdf]
