# Supplementary material for: Fecal microbiota transplantation against intestinal colonization by extended spectrum beta-lactamase producing Enterobacteriaceae: a proof of principle study
Source: BMC Res Notes. 2018 Mar 22;11:190. doi: 10.1186/s13104-018-3293-x (PMC5863815; doi:10.1186/s13104-018-3293-x)
Supplement: Supplementary file 6 — Additional file 6: Table S3. Abundances of taxa in responders vs nonresponders. Relative abundances of significantly different taxa (only < 0.9 or > 1.1 fold shown) between responders and nonresponders at baseline. Median relative abundance (percentage) is shown, also the ratio between the medians of responders and nonresponders and the p-value are given. [file 13104_2018_3293_MOESM6_ESM.doc]

Supplementary table 3

| **Taxon** | **abundance**  **in responders** | **abundance in nonresponders** | **ratio** | **p-value** |
| --- | --- | --- | --- | --- |
| **Dorea formicigenerans et rel.** | 0.0453 | 0.0277 | 1.6 | 0.035 |
| **Oxalobacter formigenes et rel.** | 0.0015 | 0.0011 | 1.4 | 0.019 |
| ***Roseburia inulinovorans*** | 0.0132 | 0.0086 | 1.5 | 0.014 |
| ***Clostridium orbiscendens*** | 1.5422 | 0.7358 | 2.1 | 0.035 |
| **Uncultured bacterium LCLC63** | 0.0167 | 0.0132 | 1.3 | 0.035 |
| **Clostridium orbiscindens et rel.** | 0.0189 | 0.0552 | 0.3 | 0.014 |

**Supplementary table 3:** Relative abundances of significantly different taxa (only <0.9 or >1.1 fold shown) between responders and nonresponders at baseline. Median relative abundance (percentage) is shown, also the ratio between the medians of responders and nonresponders and the p-value are given.
